# Identification of crucial genes involved in thyroid cancer development

**DOI:** 10.1186/s43046-023-00177-0

**Published:** 2023-05-22

**Authors:** Iyshwarya Bhaskar Kalarani, Ganesan Sivamani, Ramakrishnan Veerabathiran

**Affiliations:** 1 Human Cytogenetics and Genomics Laboratory, Faculty of Allied Health Sciences, Chettinad Hospital and Research Institute, Chettinad Academy of Research and Education, Kelambakkam, Tamilnadu 603103 India; 2 PG & Research Department of Zoology and Biotechnology, AVVM Sri Pushpam College, Poondi, Thanjavur, 613 503 Tamil Nadu India

**Keywords:** Aging process, Genetics, Molecular mechanisms, Pathogenesis, Thyroid carcinoma

## Abstract

**Background:**

A malignancy of the endocrine system, one of the most common types, is thyroid cancer. It is proven that children who receive radiation treatment for leukemia or lymphoma are at a heightened risk of thyroid cancer due to low-dose radiation exposure throughout childhood. Several factors can increase the risk of thyroid cancer (ThyCa), such as chromosomal and genetic mutations, iodine intake, TSH levels, autoimmune thyroid disorders, estrogen, obesity, lifestyle changes, and environmental contaminants.

**Objectives:**

The study aimed to identify a specific gene as an essential candidate for thyroid cancer progression. We might be able to focus on developing a better understanding of how thyroid cancer is inherited.

**Methods:**

The review article uses electronic databases such as PubMed, Google Scholar, Ovid MEDLINE, Embase, and Cochrane Central. The most frequently associated genes with thyroid cancer found on PubMed were *BAX, XRCC1, XRCC3, XPO5, IL-10, BRAF, RET*, and *K-RAS*. To perform an electronic literature search, genes derived from DisGeNET: a database of gene-disease associations, including PRKAR1A, BRAF, RET, NRAS, and KRAS, are used.

**Conclusion:**

Examining the genetics of thyroid cancer explicitly emphasizes the primary genes associated with the pathophysiology of young and older people with thyroid cancer. Developing such gene investigations at the beginning of the thyroid cancer development process can identify better outcomes and the most aggressive thyroid cancers.

## Introduction

**Fig. 1:**
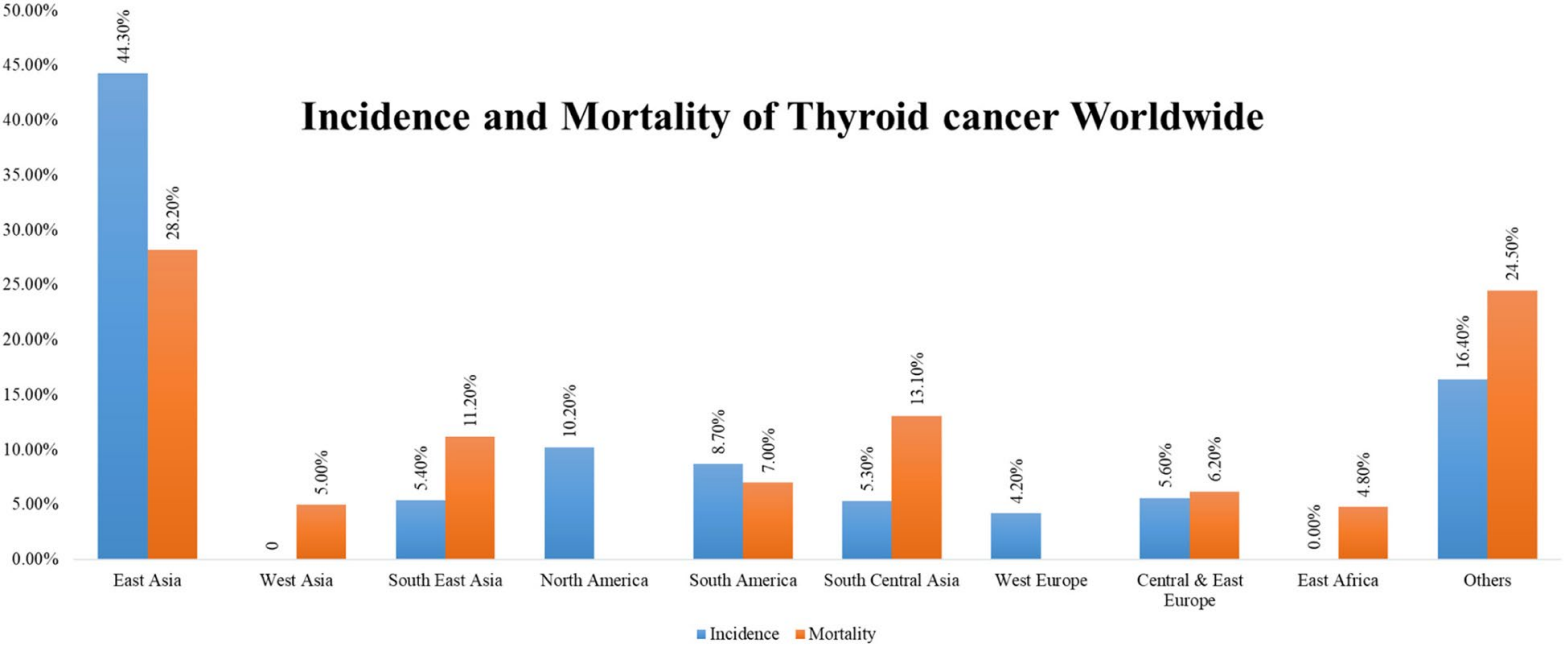
Incidence and mortality rate of thyroid cancer

Thyroid cancer (ThyCa) develops in the thyroid gland (TG), a component of the endocrine system that controls hormone production in the body by collecting iodine from the circulation and converting it into thyroid hormones, which regulate metabolism. In the USA, we primarily see it in female patients, making up 3.1% of newly diagnosed malignancies. Every year in the USA, there are around 53,990 newly diagnosed ThyCa cases and 2060 fatalities. They found ThyCa in 14.3 people per 100,000 [1–3]. It was shown that radiation exposure increased the incidence of thyroid cancer by double [4] after the World War II atomic bombs of Hiroshima and Nagasaki. There is evidence that low-dose radiation exposure throughout childhood, such as in patients undergoing therapeutic radiation for leukemia or lymphoma, is linked to an elevated risk of ThyCa [5]. There is additional evidence that children treated with low-voltage radiation for acne have an increased risk of ThyCa. Although ThyCa is more common after radiation exposure, the biological activity of the illness is identical in radiation-exposed and non-radiation-induced thyroid cancers. The concept that different types of genomic instability might lead to distinct molecular carcinogenic pathways is now widely accepted [6]. The metabolic pathways of cancer cells are also changed to provide a constant supply of components essential for synthesizing membranes and expressing genes and proteins [7]. As a result, whereas radiation exposure appears to be significant in initiating the disease, there is no evidence that it is related to cancer aggressiveness [8]. Papillary carcinoma (accounting for more than 85% of cases), followed by follicular carcinoma (5 to 15% of cases), medullary carcinoma (5% of cases), anaplastic (undifferentiated) carcinoma (< 5% of cases) [9]. Both incidence and mortality (Fig. 1) estimates were presented by country and aggregated across the 20 UN-defined world regions and according to the UN’s four-tier Human Development Index (ie, low, medium, high, and very high) in 2020 [10].

### Thyroid carcinoma: molecular pathogenesis

Carcinogenesis is a multistage process with at least three phases or stages: initiation, promotion, and progression [11]. Several pathological factors associated with functional dedifferentiation led to thyroid cancer cells having lower iodide capacities and inadequate iodine absorption. Thyroid-stimulating hormones in thyroid cancer can increase iodine absorption. Iodine metabolism is abnormally affected by thyroglobulin synthesis defects, leading to malignant thyroid cell development. Iodoproteins are also produced and released into the bloodstream by thyroid tumors [12].

**Fig. 2:**
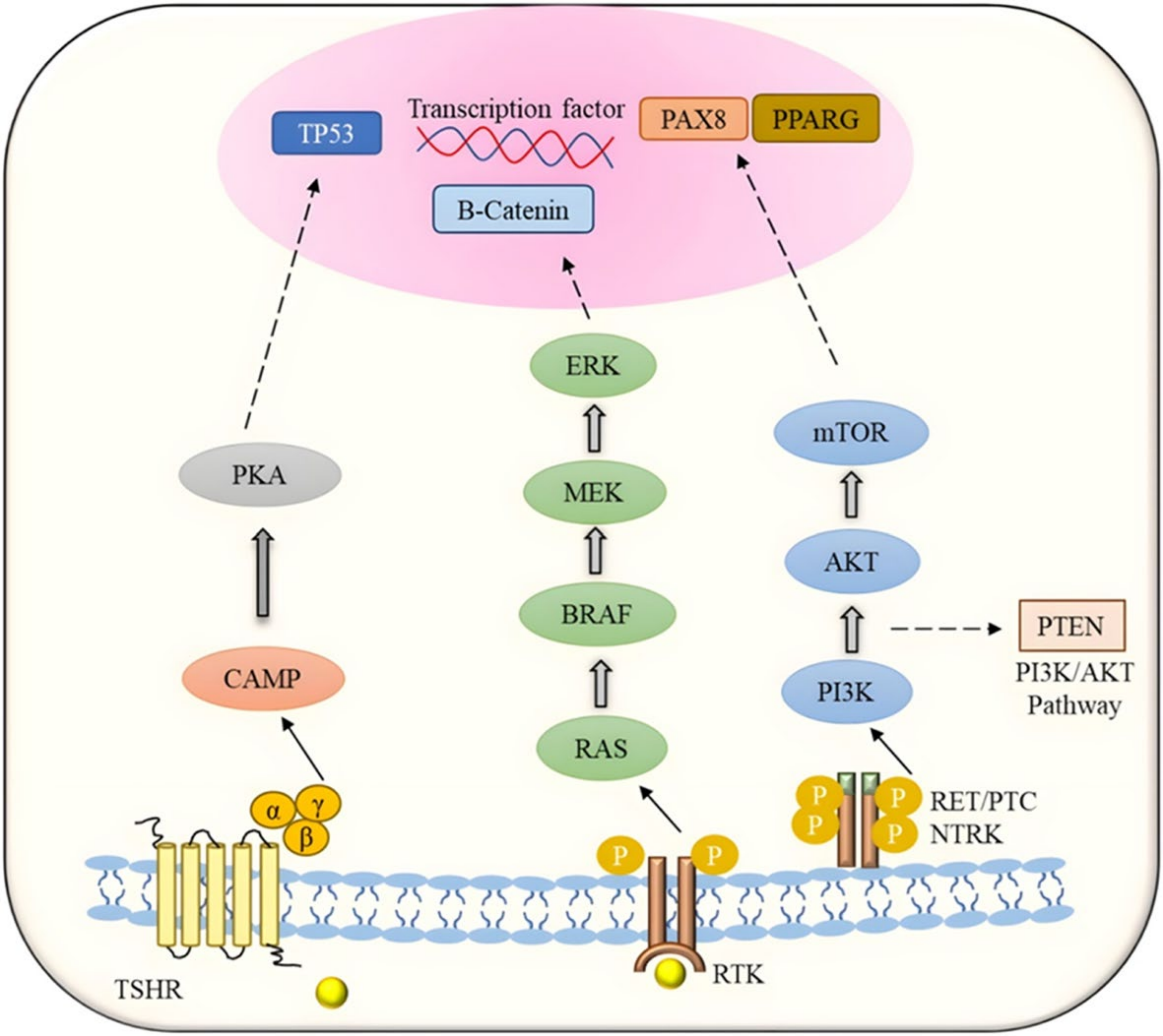
Pathogenesis of thyroid carcinoma

Over 90% of ThyCa patients have one or more genetic abnormalities [13]. Two distinct signaling cascades are involved in most of the targeted genes: Phosphatidylinositol 3-kinase (PI3K) and extracellular regulated kinase (ERK) play similar roles [14]. In eukaryotes, the ERK pathway is a numerous mitogen-activated protein kinase (MAPK) control module [15]. When a growth factor is activated, it attaches to the corresponding membrane receptor, which has tyrosine kinase catalytic activity (RTK). As a result, the RTK’s kinase activity is activated, followed by the conscription of guanine nucleotide exchange factors (GEF) that facilitates the packing of RAS small GTPases with GTP nucleotides. Besides being recruited to GTP-bound RAS, ARAF, BRAF, and CRAF, serine/threonine RAF kinases are triggered by phosphorylation and dimerization of MEK proteins, as illustrated in Fig. 2. MEKs are dual-activity kinases that phosphorylate the ERKs p44 and p42, which are serine/threonine kinases. Finally, active ERKs target a range of transcription factors directly or indirectly through other downstream kinases like p90RSK to significantly impact cellular transcriptional output [16]. RTKs or RAS, like the MAPK system, instantly activate PI3K [14]. The PIP3 trisphosphate is a phosphorylated form of phosphatidylinositol (3, 4, 5) and is the second intracellular messenger produced in PI3K. PIP3 activates the enzyme PDK1, which phosphorylates the serine/threonine kinase AKT/threonine PKB’s 308 residues.

By hydrolyzing PIP3, and PTEN (phosphatase and tensin homolog), phosphatase acts as a gatekeeper. Through inhibiting the tuberous sclerosis complex 2 (TSC2) by active AKT, mTOR rapamycin activates mTORC1’s mammalian target, initiating the Rheb small GTPase. mTOR promotes in a cell in which protein synthesis takes place and proliferation. The eukaryotic translation initiation factor 4E-binding protein 1 (4E-BP1) is phosphorylated by p70-S6 kinase, which phosphorylates the ribosomal protein S6. Genetic mutations target several PI3K signaling pathway components in ThyCa [17, 18].

### Thyroid tumor genesis and aging processes

Women are two to three times as likely as men to be affected by papillary thyroid cancer (PTC). The female-to-male ratio appears to be declining with age [19]. Notably, the elderly have a greater death risk from PTC. This is probably due to the higher mitotic activity of these tumors and the possibility of distant metastases [20]. Patients with aggressive PTC variations are more likely to gain metastatic disease in the general population identified [21]. The second most prevalent thyroid cancer is follicular thyroid carcinoma (FTC). Compared to PTC, this cancer has a poorer prognosis since it is more prone to hematogenous spread to distant areas. Up to 5% of thyroid malignancies are medullary thyroid carcinomas (MTC), which develop from the thyroid gland's parafollicular cells (C cells). Its sporadic form is more common than familial MTC and affects older adults more frequently. Anaplastic (undifferentiated) thyroid cancer (ATC) that proliferates and is often highly aggressive is uncommon; it should be emphasized, however, that elderly individuals are more likely to have it than younger ones. Most individuals have extensive local invasion and distant metastases by diagnosis. ATC’s poor prognosis appears to be strongly correlated with age [22].

### Younger and older age population in thyroid carcinoma

Most cases in the 20- to 39-year-old age group are differentiated thyroid carcinoma (DTC), with PTC being the most common, accounting for around 85% of thyroid malignancies. Among young adults, the age group of 35 to 39 years old has the highest frequency of DTC. It is substantially more prevalent in females than males, and young adults, like the overall population, have seen a considerable increase in thyroid cancer incidence [23]. Thyroid cancer comprises various subtypes, but MTC is a rare subtype representing approximately 5% of all thyroid cancers [24]. Only around one-third of MTC cases involve people under 40, with a median age of diagnosis of 50.

Young individuals aged 20 to 24 have an annual incidence rate of 0.6 cases per million, whereas those aged 35 to 39 have a yearly incidence rate of 1.5 cases per million. In contrast to DTC, the young adult population shows no overt gender preference, with an average of 1.6 males for every female [25]. One in three young people, in addition to familial MTC, there is a familial multi-endocrine neoplasia type 2a or type 2b which are inherited tumor syndromes that cause familial MTC, are impacted (FMTC). Besides MTC, pheochromocytomas and hyperparathyroidism can develop in up to 20% of MEN2A patients and 50% of MEN2A and MEN2B patients. Additional symptoms include Hirschsprung’s disease, PTC, and cutaneous lichen amyloidosis [26]. Younger thyroid cancer patients outlived older patients overall and in cancer-specific survival, but they may receive more aggressive treatment [27]. Although many aging-related illnesses appear considerably more frequently in both age groups, there is a higher risk of thyroid cancer for thyroid cancer survivors diagnosed before the age of 40 [28]. We believe that one of the best indicators of prognosis is the age at which diagnosed anaplastic thyroid carcinoma is. Kim et al. investigated one hundred twenty-one patients with anaplastic thyroid carcinoma. In a multivariate analysis, age under 60, tumor size under 7 cm, and disease severity were independent predictors of a decline in cause-specific mortality [29]. Female MTC patients under 45 with thyroid involvement had the best overall prognosis of 100% survival after 10 years [30]. With a mean diagnosis of about 47 years, the sporadic variation of medullary carcinoma is more common in older people than the genetic variant in younger people. It can pass the hereditary form down through families. This condition occurs either as a genetic condition or as an expression of type 2A or type 2B multiple endocrine neoplasia syndrome [31].

### Age and thyroid cancer staging: implications for prognosis

Risk stratification is one of the essential aspects of DTC treatment. Upon diagnosis, the clinicopathologic features of the tumor and the patient’s age are used to determine the prognosis. They associated a lower chance of survival in older patients with their age at diagnosis. The American Joint Committee on Cancer staging approach has the age in it DTC as the sole human malignancy. The patient’s age is a reliable indicator of ThyCa mortality, albeit the reason is unknown. The occurrence of the connection suggests that either the disease or the treatment has an age-dependent component [32, 33]. Research has shown that differences in the sodium–iodide symporter expression, essential for radioiodine absorption, correlate with patient age [34–36]. Age seems to affect how well a patient responds to therapy. Shah et al. examined death and recurrence rates in high-risk groups by American Thyroid Association (ATA), a risk assessment organization [37]. Patients over 55 had less chance of benefiting from therapy than younger patients. The disease-specific survival was lower in older patients with a partial response to treatment in the ATA-high-risk category. It has been found that tumor volume, type, penetrating vascular, extrathyroidal tumor extension, metastatic infection, and neck examination performance were all independent predictors of partial treatment response.

A molecular study has clarified the relevance of numerous tumor mutations, especially in the DTC oncogenesis, and the relationship with prognosis and age, BRAF, and TERT is essential [38–42]. The BRAF mutation has been traditionally about 45% of PTC patients in the USA have this condition, which is connected to reduced thyroid differentiation indicators such as thyroglobulin, thyroid peroxidase, and sodium/iodide symporter. Mutations in a PTC tumor are more likely to exist [43–45]. Although the BRAF mutation is a diagnostic marker for PTC, it is not a reliable sign of PTC mortality or recurrence [46, 47]. It has proven TERT promoter mutations reliable indicators of cancer-related mortality and DTC recurrence [48, 49]. Among the men and women living in the United States, there is a gender disparity in thyroid cancer incidence primarily confined to small subclinical PTCs detected. As a result of this trend, women have been affected more than men: during the period 1975 to 2017, PTC incidence increased by 13.3 cases per 100,000 women (from 4.6 to 17.9 cases, or 389%), while it decreased by 4.3 cases per 100,000 men (from 2.2 to 6.5 cases, or 295%). Several factors, including hormonal and reproductive factors, have been suggested to increase the likelihood of women developing thyroid cancer over men. There have been suggestions that thyroid cancer may be linked to a recent pregnancy, infertility, abnormal menstruation cycles, and breast cancer [50].

### Genes associated with thyroid carcinoma

**Fig. 3:**
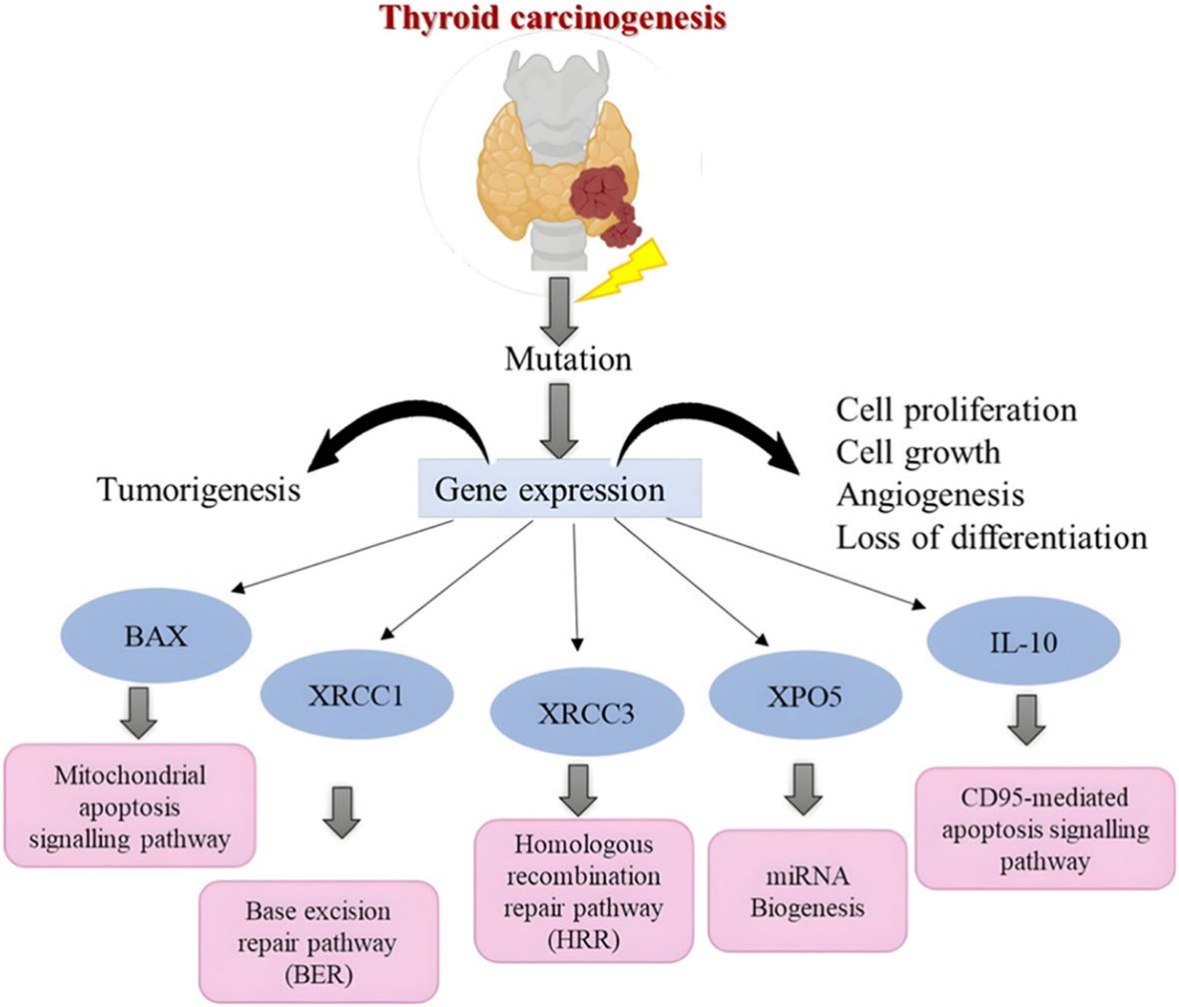
Genetic basis of thyroid carcinoma

Different thyroid cancer types have been associated with changes in some genes and pathways, as shown in Fig. 3. This review summarises the most relevant ones (Table 1). DNA repair genes can be affected by gene polymorphisms that result in amino acid substitutions, resulting in different capacities to repair DNA damage due to genetic polymorphisms associated with amino acid substitutions. There have been several studies that have shown that certain types of environmental pollution are associated with genetic instability and the development of cancer. There have been several types of DNA repair mechanisms, including base excision repair (BER), nucleotide excision repair (NER), double-strand break repair (DSBR), mismatch repair (MR), and homologous recombination repair (HRR) that have been identified in mammalian cells. Ionizing radiation or oxidative damage in combination with methylation, oxidation, or reduction of non-bulky base adducts serves as the trigger for the BER pathway to eliminate them. As part of maintaining genomic integrity, several DNA repair pathways are regulated carefully, as well as modulating the repair capability in response to DNA damage to modulate TC susceptibility [51].

**Table 1:** Candidate gene associated with thyroid carcinoma

S.no	Gene symbol	Gene name	Chromosomal location	Exon	rsID	Function	References
1	*BAX*	BCL-2 associated X	19q13.11	7	248 G > A	P53 regulates apoptosis mediated by P53 and necessitates the expression of this gene	[52]
2	*XRCC1*	X-ray repair cross-complementing group 1	19q13.31	17	rs25487, rs25489 rs1799782	This enzyme may control Meiogenesis and recombination within germ cells	[53]
3	*XRCC3*	X-ray repair cross-complementing group 3	14q32.3	11	rs861539	This gene has been associated with cancer in patients who are radiosensitive and nonradiosensitive	[54]
4	*XPO5*	Exportin 5	6p21.2	33	rs11077	An essential transporter of small RNAs and double-stranded RNAs, Karyopherin is the protein encoded by this gene	[55]
5	*IL-10*	Interleukin 10	1q31-32	7	IL-10–1082 G allele	Th1 cytokines, MHC class II Ags, and macrophage stimulation molecules are down-regulated by this substance. It also promotes antibody survival, proliferation, and production in B cells	[56]
6	*BRAF*	B-Raf Proto oncogene	7q34	24	–	It is a protein kinase that transduces mitogenic signals from the membrane to the nucleus of cells	[57]
7	*RET*	Ret Proto Oncogene	10q11.21	20	–	When bound to ligands from the glial cell-derived neurotrophic factor family, this receptor tyrosine-protein kinase facilitates the growth of glial cells, cell navigation, cell migration, and cell differentiation	[58]
8	*K-RAS*	K-RAS Proto Oncogene	12p12.1	7	–	Regulates cell proliferation by playing an important role	[59, 60]

A wide variety of diseases are associated with aberrant miRNA expressions, including cancer, according to a recent study that suggests that miRNA expressions may be related to a wide range of diseases. Several previous studies have demonstrated that the dysregulation of miRNAs in TC significantly affects the expression. The most important biological processes are imperative for survival are proliferation, metastasis, invasion, and apoptosis. When overexpression of XPO5 is observed, it is believed that the activity of miRNAs will be increased, whereas a decrease in XPO5 expression is thought this compound inhibits Pre-miRNA export; as a result, the increase in miRNA activity. The study of miRNA activity and quantity has demonstrated that even a slight alteration can adversely affect target mRNAs and cellular functions, so miRNA-related single-nucleotide polymorphisms have been identified in recent years as potential and valuable biomarkers for cancer prediction and prognosis [61].

It is critical the understanding the tumor microenvironment to be aware of how cancer occurs and develops. Several components, such as immune cells, stromal cells, cytokines, and chemokines, are involved in the immune system that can influence tumor growth positively or negatively. T lymphocytes, monocytes, macrophages, and endothelial cells are significant sources of interleukin production. Interleukin is a small protein signaling molecule in the cytokine family most commonly produced by these cells. Several studies have demonstrated that interleukins, such as IL-1 and IL-38, play a significant role in developing various types of cancer, such as breast, hepatocellular, and thyroid cancers [57]. Gene changes result from genetic modifications that encode cell signaling pathways contributing to tumor transformation by causing imbalances in the relationship between proliferation and apoptosis in cells. Specific gene mutations have been linked to metastatic lymph node spread due to increased tumor aggressiveness, a tendency to dedifferentiate, and reduced efficiency of radioiodine treatments. We selected these genes for this study based on this concept.

### BCL-2 associated X

A crucial member of the Bcl-2 family of antiapoptotic proteins is B cell lymphoma 2 (Bcl-2), linked with protein X. The BAX gene is located at position 19q13.33. and contains seven exons, which are primarily found in the cytoplasm. The apoptotic factor cytochrome C may also be produced by BAX proteins, enhancing the susceptibility of the mitochondrial membrane and starting the apoptosis cascade reaction. There is an antiapoptotic protein called Bcl-2 present in most thyroid tumors, including follicular neoplasms (FN), papillary carcinomas (PTC), and medullary carcinomas (MTC), but not in most undifferentiated thyroid tumors. It is the overexpression of Bcl-2 which is associated with poor prognosis. It has been shown that Bcl-2 plays a role in thyroid cancer. A decrease in Bcl-2 was associated with a loss of differentiation ability in thyroid tumors [62]. BAX is thus required for regulating the mitochondrial apoptosis signaling pathway [63]. Polymorphisms in the BAX gene and cancer depend on the patient’s prognosis and the prevalence of the disease. It is a decreased amount of specific protein produced and thought to be caused by the (248 G > A) polymorphism, while it connected this to the transcriptional activity of the BAX gene being negatively regulated. This increases the Bcl-2 to BAX proportion and could inhibit apoptosis in tumor cells [64]. Over 70% of differentiated carcinomas have molecular markers for thyroid cancer, and knowledge of its many molecular pathways opens up new avenues for its detection and therapy [52]. The genetic variants, which are molecular changes in at least 1% of the population and they refer to as SNPs when they occur in only one nucleotide, are among the different molecular markers known as SNPs (single nucleotide polymorphisms). The BAX SNP (248 G > A) (rs4645878) genotype and allele frequencies are to be linked to a greater risk of PTC [65].

### X-ray repair cross-complementing group 1

Chromosome 19q13.31 has the 17 exon XRCC1 gene. In the BER pathway, this protein collaborates with DNA ligase III, DNA polymerase, and PARP to repair single-strand DNA breaks [66, 67]. Base excision repair (BER) and single-strand break repair (SSBR) depend significantly on them. In the presence of reactive oxygen species produced by the body, ionizing radiation or alkylating agents [68, 69]. BER predominantly removes it based on base adducts that are not bulky and are created by methylation, oxidation, and reduction [53]. A recent study has clarified the biological importance of frequent XRCC1 polymorphisms (rs25487, rs25489, and rs1799782). However, genetic investigations are still unclear in this area [70]. As previously shown by research [71, 72], There may be a link between DTC risk and XRCC1-rs1799782 in the Chinese population. Other investigations have found that the XRCC1-rs1799782 polymorphism has little effect on DTC susceptibility in different ethnic groups [73]. The SNP rs25489 has, however, been linked positively to DTC risk in the Caucasian population [74]. To coordinate the rate and sequence of enzymatic activities and prevent the release of harmful DNA intermediates into the cellular environment, XRCC1 interacts with various enzymes and DNA intermediates in different DNA repair pathways [66].

### X-ray repair cross-complementing group 3

The RAD51 gene encodes a protein belonging to the RecA/Rad51 family. To preserve chromosomal integrity and repair DNA damage brought on by endogenous and exogenous sources, and is structurally and functionally connected to the XRCC3 gene on chromosome 14q32.3 [75, 76]. One of the most crucial proteins in the homologous recombination repair process (HRR). It interacts with and stabilizes RAD51, contributing to HRR for DNA double-strand breaks (DSBs) and cross-link repairs in eukaryotic cells [77]. According to earlier research, the XRCC3-rs861539 polymorphism impacts DNA repair capacity, which is linked to the risk of developing cancer [54, 78]. According to an earlier study, the XRCC3-rs861539 polymorphism affects DNA repair capacity and may be linked to cancer risk. Future research is still needed to fully understand the relationship between DTC risk and the four XRCC3 SNPs (rs861539, rs1799794, rs56377012, and rs1799796), though [79].

### Exportin 5

The nucleocytoplasmic transport protein exportin (XPO5) and members of the importin-b family on chromosome 6p21.1 are well-known regulators of siRNA and miRNA nuclear export. Because XPO5 is a Ran-guanosine triphosphate (GTP)-dependent dsRNA-binding protein, the pre-miRNAs are delivered into the cytoplasm through a GTP-dependent mechanism. The pre-miRNAs mature in the cell after export before becoming functional miRNAs [55, 80]. Researchers have also discovered that XPO5 (rs11077) may protect pre-miRNAs from nuclear degradation [81]. The decreased expression of miRNAs that results from XPO5 deletion may contribute to cancer development, progression, and metastasis. According to Jing and colleagues’ research, XPO5 expression varies across healthy and tumor tissues and decreases in malignancies. ThyCa may have been formed because XPO5 inhibits the growth of tumors in ThyCa cells. Tests also confirmed their findings on hepatocellular carcinoma and colorectal cancer cells [61, 82].

### Interleukin-10

The Interleukin**-**10 genes are found at 1q31 to 1q32 on chromosome 1 and comprise five exons. It is an anti-inflammatory cytokine. As a growth factor, it stimulates the humoral immune response by activating T and B cells, monocytes, and thymocytes. Many of the pathogenic properties of thyroid cancer can be attributed to the production of IL-10 by thyroid cancer cells. IL-10 promotes the survival and proliferation of thyroid cancer cells [83]. Studies have linked SNPs in the promoter region–174 to prostate, colorectal, and pancreatic cancers [84], and other studies have found conflicting results [85]. The genetic risk factor for PTC is thought to be the IL-10- 1082 polymorphism [56]. The IL-10–1082 G allele and the GG genotype, connected with increased IL-10 production, were more common in PTC patients. Once age, sex, and smoking status are controlled, beings with the IL-10–1082 GG genotype are twice as likely to develop thyroid cancer as those with the AA genotype [83]. Finally, they assert that PTC may be linked to the IL-10–1082 G allele. The IL-10 gene variant may significantly impact the pathophysiology of PTC, which may also lead to the development of new medicinal techniques. When it is discovered, high-risk individuals may be subjected to more rigorous examinations to diagnose minor PTC. A high-risk group for PTC can also be designed with personalized treatment and preventive strategies [84]. IL-10 might promote thyroid cancer aggressiveness by suppressing the immune system and promoting immune escape from thyroid cancer cells [57].

### B-Raf proto-oncogene

**Fig. 4:**
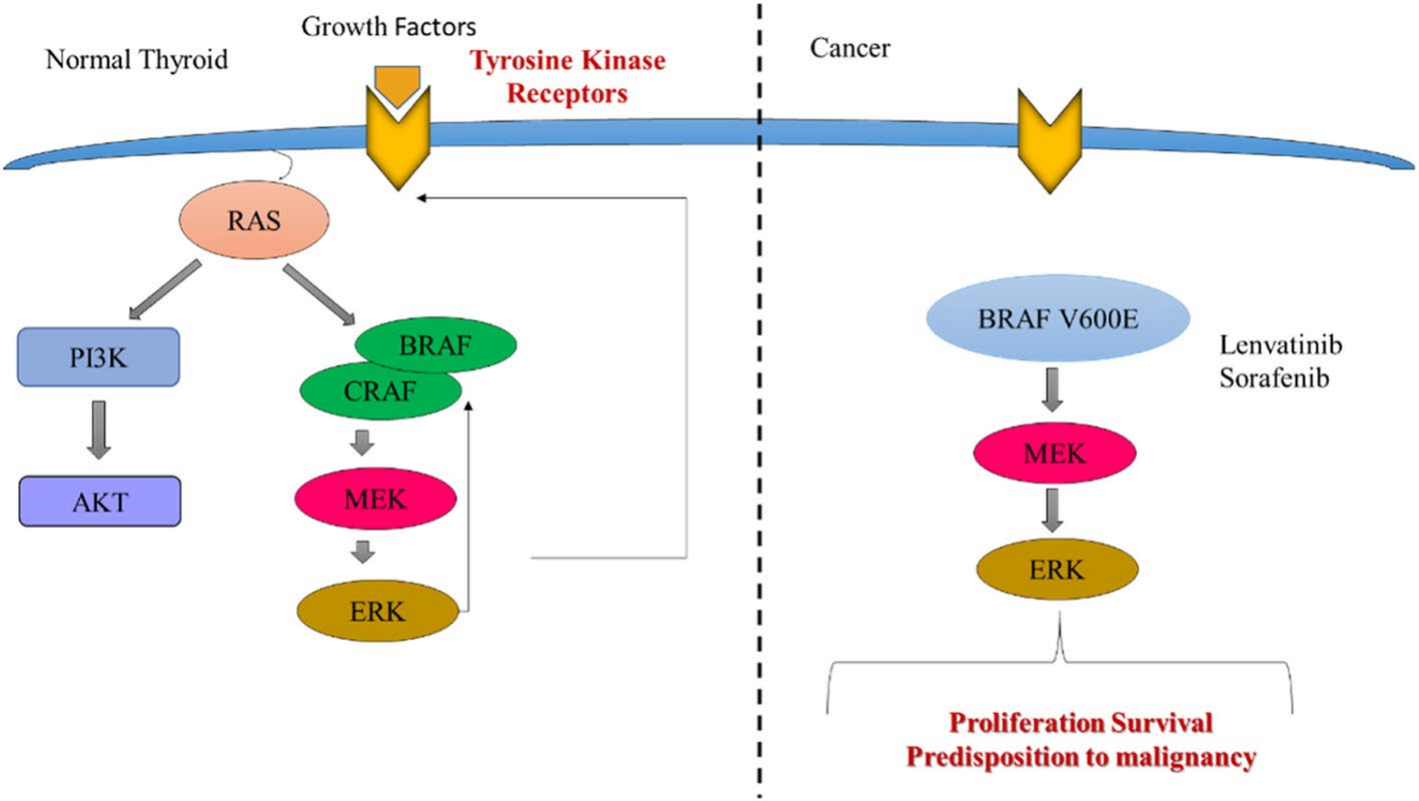
BRAF^V600E^ signaling in thyroid carcinoma

In addition to being a proto-oncogene, BRAF plays a crucial role in regulating cell proliferation, differentiation, and programmed cell death [86]. Most thyroid carcinomas are caused by point mutations (BRAF V600E) occurring at codon 600 of the 15th exon of the BRAF gene [87]. This mutation permanently activates the BRAF protein. Several genes are impacted by the mutant BRAF protein, including those for thyroglobulin, thyroperoxidase, and others. The NIS (natriumiodide symporter) gene expression is reduced, as are the genes for thyroglobulin and thyroperoxidase [88, 89]. As a result of this mutation, several studies have linked this mutation to a worse prognosis, a higher likelihood of recurrence, and an increase. There is evidence of decreased iodine transport into the cells, increased tumor aggressiveness, extrathyroidal spread, and local and distant lymph node metastases [90]. As a result of having the BRAF V600E mutation, there is a nearly 100% chance that you will develop cancer in the future [58]. The oncogenic BRAFV600E activates the MAPK pathway independently of extracellular stimuli. As a result, there is no negative regulation from ERK to RAF dimerization, leading to strong activation of the pathway (Fig. 4) [91].

### Ret proto-oncogene

A transmembrane tyrosine kinase receptor encoded by the RET proto-oncogene is critical to cell proliferation, differentiation, and survival. RET gene mutations are commonly found in medullary carcinomas (MTCs) due to point mutations in the gene [92]. More than 95% of the patients with MEN2A and MEN2B were found to have genetic alterations, compared with 50% of the patients who had known MTC and over 95% with sporadic MTC [93]. Family members of patients with a detectable germline mutation in the RET gene are recommended to undergo genetic testing [41]. There is a significant risk of MTC associated with an inherited RET mutation. Therefore, a complete preventative thyroidectomy is recommended for patients with an inherited RET mutation [52, 59]. As a result of the genotype–phenotype correlations that have been found, individual recommendations are created, particularly regarding scheduling a complete preventive thyroidectomy in childhood to prevent thyroiditis from developing [60]. In thyroid cancers, there is a rearrangement of the tyrosine kinase receptor. MAPK and PI3K downstream pathways are activated by binding RET, NTRK, and ALK agonists in normal thyroid tissue [91].

### K-RAS proto-oncogene gene

**Fig. 5:**
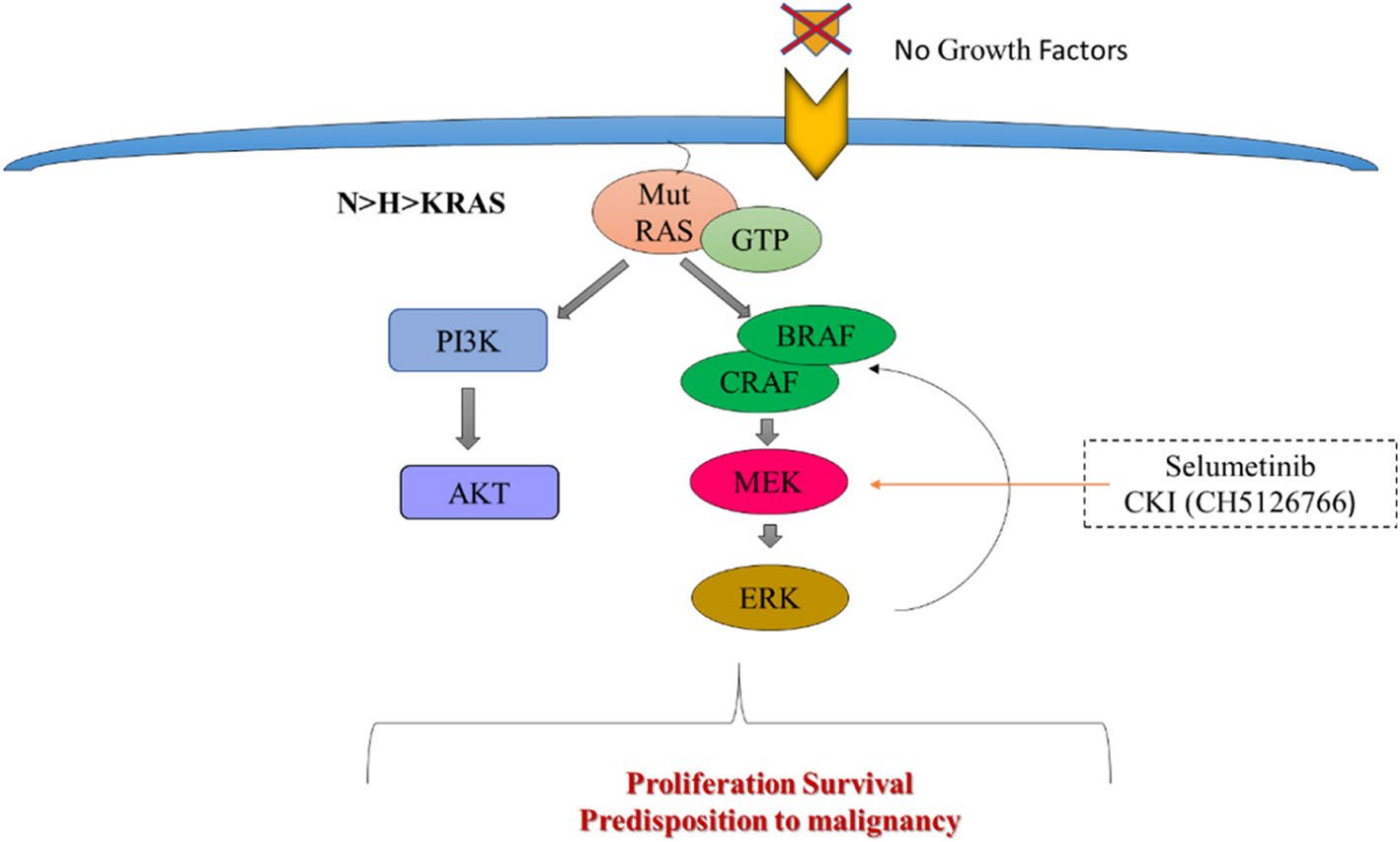
Impact of *RAS* mutations in thyuroid carcinomas

In addition to acting as signal transducers between transmembrane tyrosine kinase receptors and the nucleus, these proteins contribute to cell growth and differentiation via the MAPK or PI3K-AKT pathways (Fig. 5). A proto-oncogene can transform into an oncogene when a point mutation occurs in one of these genes. Oncogenes stimulate cell proliferation while inhibiting differentiation. A common mutation in thyroid tumors results in modifications in the HRAS, KRAS, and NRAS genes, and it is still unclear what their significance is. Both benign and malignant thyroid tumors can contain mutations in these genes. A mutation in the RAS gene may play a role in the progression of a benign tumor to carcinoma in the future [92, 94]. PTCs arising from follicular variants are found to have RAS mutations in approximately 20% of cases [86, 95]. The thyroid biopsy is most commonly used to detect these genetic changes and BRAF mutations in the human body. ETA recommends performing less radical surgery when pathogenic mutations in RAS genes are detected (e.g., hemithyroidectomy) while considering the patient's clinical and anamnestic history [96].

## Conclusion

As we age, our endocrine system, including the thyroid gland, changes how it regulates biological functions. Establishing a physiological norm for thyroid hormone levels is challenging due to the increasing resetting of the hypothalamic-pituitary-thyroid axis with age. This results in elevated levels of TSH. Thyroid carcinoma is more common among older adults, and men are more prone than women to developing these tumors aggressively. The mortality risk of thyroid carcinoma increases significantly with age from the ages of 40 to 45. Thyrocytes and follicular cells, the gland’s epithelial components, are where ThyCa originate. ThyCa is divided into two types based on clinical characteristics and appearance: differentiated (which includes medullary, follicular, and papillary carcinoma) and anaplastic (which does not include medullary, follicular, or papillary carcinoma). Proliferation, survival, and tumorigenesis are all regulated by the MAPK pathway. This pathway significantly contributes to thyroid tumorigenesis, particularly in PTC. MAPK pathway activators are responsible for driving thyroid cancer. This review highlights that although the link between age and thyroid cancer is still not obvious, generation continues to be a significant predictive factor for thyroid cancer. They were identifying these critical genes as potential biomarkers improves thyroid cancer patients’ early diagnosis and survival. By developing such gene investigations at the beginning of the thyroid cancer development process, we can achieve better outcomes and identify the most aggressive forms of thyroid cancer. Finally, altered thyroid function may significantly influence lifetime control via several pathways. Reduced thyroid function, in particular, may contribute to an increased lifespan.

## Data Availability

Not applicable.

## Abbreviations

ThyCa: Thyroid cancer
TSH: Thyroid-stimulating hormone
AITD: Autoimmune thyroid disease
TG: Thyroid gland
ERK: Extracellular regulated kinase
PI3K: Phosphatidylinositol 3-kinase
MAPK: Mitogen-activated protein kinase
RTK: Tyrosine kinase catalytic activity
GEF: Guanine nucleotide exchange factors
PTC: Papillary thyroid cancer
FTC: Follicular thyroid cancer
MTC: Medullary thyroid cancer
ATC: Anaplastic thyroid cancer
DTC: Differentiated thyroid cancer
PRKAR1A: Protein kinase cAMP-dependent type I regulatory subunit alpha
BRAF: B-Raf proto-oncogene; serine/threonine kinase
RET: Ret proto-oncogene
NRAS: NRAS proto-oncogene; GTPase
